# Epigenetic Transgenerational Inheritance of the Effects of Obesogen Exposure

**DOI:** 10.3389/fendo.2021.787580

**Published:** 2021-12-16

**Authors:** Nicole Mohajer, Erika M. Joloya, Jeongbin Seo, Toshi Shioda, Bruce Blumberg

**Affiliations:** ^1^ Department of Pharmaceutical Sciences, University of California, Irvine, CA, United States; ^2^ Department of Developmental and Cell Biology, University of California, Irvine, CA, United States; ^3^ Center for Cancer Research, Massachusetts General Hospital, Charlestown, MA, United States; ^4^ Department of Biomedical Engineering, University of California, Irvine, CA, United States

**Keywords:** epigenetics, transgenerational, obesogen, endocrine disrupting chemicals, transgenerational inheritance, obesity, EDC

## Abstract

Obesity and metabolic disorders have become a worldwide pandemic affecting millions of people. Although obesity is a multifaceted disease, there is growing evidence supporting the obesogen hypothesis, which proposes that exposure to a subset of endocrine disrupting chemicals (EDCs), known as obesogens, promotes obesity. While these effects can be observed *in vitro* using cell models*, in vivo* and human epidemiological studies have strengthened this hypothesis. Evidence from animal models showed that the effects of obesogen exposure can be inherited transgenerationally through at least the F4 generation. Transgenerational effects of EDC exposure predispose future generations to undesirable phenotypic traits and diseases, including obesity and related metabolic disorders. The exact mechanisms through which phenotypic traits are passed from an exposed organism to their offspring, without altering the primary DNA sequence, remain largely unknown. Recent research has provided strong evidence suggesting that a variety of epigenetic mechanisms may underlie transgenerational inheritance. These include differential DNA methylation, histone methylation, histone retention, the expression and/or deposition of non-coding RNAs and large-scale alterations in chromatin structure and organization. This review highlights the most recent advances in the field of epigenetics with respect to the transgenerational effects of environmental obesogens. We highlight throughout the paper the strengths and weaknesses of the evidence for proposed mechanisms underlying transgenerational inheritance and why none of these is sufficient to fully explain the phenomenon. We propose that changes in higher order chromatin organization and structure may be a plausible explanation for how some disease predispositions are heritable through multiple generations, including those that were not exposed. A solid understanding of these possible mechanisms is essential to fully understanding how environmental exposures can lead to inherited susceptibility to diseases such as obesity.

## Epigenetics

The British embryologist, Conrad Hal Waddington has been credited with coining the term “epigenetics” after his discovery that genetic assimilation drives phenotypic evolution (1, 2). Epigenetics is the study of heritable changes in gene expression that are “on top of genetics”; i.e., those that change gene expression without changing the nucleotide sequence of the genome itself. Upon exposing a primary generation of fruit flies to ether, Waddington discovered that mating the bithorax mutants would result in the phenotype being inherited over multiple generations that were not treated with the chemical (3). He described this phenomenon as genetic assimilation through canalization, which essentially means that the phenotype is biologically robust despite possible genetic or environmental variations. Barbara McClintock’s groundbreaking studies using corn revealed that a gene located on chromosome 9 that was responsible for chromosome breakage [the dissociation (Ds) locus], had the ability to move locations along the chromosome and could even end up on other chromosomes. She called this a “jumping gene” and showed that its movement to different parts of the genome resulted in different phenotypes. The proximity of the jumped gene to other genes allowed it to alter the expression of those nearby genes without changing the DNA sequence (4). Since the discovery of epigenetics to present day, the phenomenon has been studied in both animal and plant models, shedding light on how normal development and endocrine homeostasis can be altered in future generations without disrupting nucleotide sequence.

## Transgenerational Inheritance

Multiple environmental factors, such as exposure to stress, toxicants or altered nutrition have been shown to create epigenetic changes that are passed down generations of offspring, some of which, are never directly exposed to those factors (5). Transgenerational inheritance of environmental exposures refers to the inheritance of a phenotype in a generation that was never exposed to the stimuli itself, *via* the germline (5). When the F0 generation is exposed to a chemical or an environmental stimulus, subsequent generations can also be exposed and/or affected through various avenues. For example, chemical exposure of an F0 female mammal during pregnancy will have direct effects on her F1 generation offspring that are exposed to the chemical *in utero*. The F2 generation is exposed to the stimuli as germ cells inside the F1 offspring and the F2 generation is still directly exposure to the stimulus. The effects observed in the F1 and F2 generations during F0 exposure are called multi- or intergenerational effects. Effects seen in the F3 generation of offspring and beyond are called transgenerational since these offspring were never directly exposed to the stimulus and can only be affected by F0 *via* epigenetic changes. If the F0 animal is male, or a non-pregnant female, then the F1 generation is exposed as F0 germ cells and the first transgenerational (unexposed) generation is the F2 offspring. In oviparous animals such as fish, reptiles, chickens, *Drosophila* and *C. elegans*, F2 is always the first transgenerational generation. While the inheritance of altered DNA sequences (e.g., mutations) is well studied, the mechanisms underlying inheritance of phenotypes in the absence of DNA sequence alterations remain poorly understood, particularly in the unexposed generations.

Evidence of transgenerational inheritance of environmental exposures has been found in multiple model organisms in different labs around the world. One type of environmental stressor, caloric restriction, was found to extend the lifespan yet decrease fertility in multiple species including *C. elegans* (6). More recent studies have explored this link between parental metabolic factors and transgenerational effects into the F3 generation of offspring. Studies with *C. elegans* have found that starvation of the parental generation led to altered metabolism in the F3 generation while a glucose enriched diet in the parental generation lead to a heritable phenotype that decreased fertility in the F1 and F2 generations (7, 8). Various transcription factors and genes involved in the insulin/IGF-signaling pathway that regulate how an organism reacts to nutritional states were mutated to further explore the pathways involved in the link between glucose enrichment and decreased progeny (such as *daf-16*, *aak-2*, and *sir-2*, and *hif-1*). However, increased glucose levels reduced progeny in all mutant groups (8). In the fruit fly, *Drosophila melanogaster*, long-term high dose exposure to the plasticizer, bis(2-ethylhexyl) phthalate (DEHP), in the F0 generation led to increased body weight of the offspring (9). In euryhaline fish, *Menidia beryllina*, exposure to EDCs such as bifenthrin, levonorgestrel and trenbolone at picomolar levels in the F0 generation during fertilization led to significant differences in promoter and gene body methylation in the F1 and F2 generations (10). Exposure of F1 generation rats to high doses of the obesogens bisphenol A (BPA), DEHP, or dichlorodiphenyltrichloroethane (DDT) led to transgenerational inheritance of obesity in the F3 generation, with over 50% of both male and female mice developing obesity (11, 12). These findings indicated that transgenerational inheritance is a real phenomenon in plants and different types of animal models. However, whether and how transgenerational epigenetic inheritance occurs in mammals is subject to debate because the underlying mechanisms remain obscure and inconclusive. This review will focus on the epigenetic mechanisms that have been suggested to play a role in the transgenerational inheritance of obesity in response to environmental obesogens. Such mechanisms include DNA methylation, histone retention, histone methylation, and changes in noncoding RNA. Finally, we propose that alterations in higher order chromatin organization may provide a unifying mechanism underlying transgenerational inheritance.

## Transgenerational Inheritance of Obesity

Obesogens were defined as chemicals that elicit increased fat mass after exposure in a living organism, whereas potential obesogens can cause adipocyte differentiation in culture or activate pathways known to be important for adipogenesis, *in vitro* [reviewed in (13)]. The well-known environmental obesogen, tributyltin (TBT) activates the nuclear receptors peroxisome proliferator activated receptor gamma (PPARγ) and its heterodimeric partner, retinoid ‘X’ receptor (RXR) (14, 15). When activated by ligands such as TBT, PPARγ and RXR form a heterodimer that promotes commitment of multipotent mesenchymal stromal stem cells (a.k.a. mesenchymal stem cells, MSCs) to the adipose lineage and differentiation of pre-adipocytes into mature adipocytes (15–17). In the early 2000’s, it was found that TBT induced isolated mesenchymal stem cells (MSCs) to become adipocytes *in vitro*, classifying it as a potential obesogen (16, 18, 19). MSCs derived from the F1 offspring of pregnant F0 dams exposed to TBT *in utero* were predisposed toward the adipogenic and against the osteogenic pathway (19).

TBT was tested in *in vivo* studies using the amphibian, *Xenopus laevis*, and in mouse models and verified to be a bona fide obesogen (15, 20). When pregnant F0 mouse dams were exposed to TBT during pregnancy (20) or throughout pregnancy and lactation (21) the F3 and F4 male offspring were found to have increased WAT when exposed to increased dietary fat. Thus, TBT exposure in the F0 generation resulted in a sex-specific transgenerational predisposition to obesity as well as a “thrifty phenotype” (21). Prenatal TBT exposure had lasting effects on both exposed (F1, F2) and unexposed (F3, F4) offspring. Offspring derived from pregnant F0 dams that were exposed to TBT or rosiglitazone exhibited increased WAT depot weight, increased numbers and size of white adipocytes (20) as well as the previously noted reprogramming of MSCs towards the adipocyte lineage (19). This was accompanied by up-regulation of genes associated with lipid storage and transport, lipogenesis and lipolysis as well as fatty livers (20).

Exposure of pregnant F0 mouse dams to bisphenol S (BPS) throughout gestation and lactation also resulted in a sex-specific obesogenic effect that impacted all generations into F3. When body weight, adipose tissue, and plasma analyses were analyzed from each generation of offspring, the F2 generation showed increased body weight, increased fat, and higher VAT mass in both males and females. However, plasma parameters differed between the genders. F3 generation males showed no effects from ancestral BPS exposure while in females, VAT mass decreased and expression of lipogenic genes increased markedly. The authors suggested that DNA methylation, histone acetylation, and/or non-coding RNAs were likely avenues of transgenerational inheritance, although these avenues were not explored in the study (22).

Below we discuss possible mechanisms through which transgenerational inheritance might occur and the evidence available in support of each. A list of obesogens thought to act epigenetically and possible mechanisms of action is presented in **Table 1**.

**Table 1:** Obesogens thought to act epigenetically and possible mechanisms of action.

*Chemical*	*Proposed mechanism*						*Model type*		*Phenotype*	*References*
	*DNA methylation*	*Histone methylation*	*Histone retention*	*ncRNA*	*Chromatin structure*	*Unknown*	*In vitro*	*In vivo*		
*Vinclozolin*	Promotes DNA Methylation Regions in offspring sperm		Promotes differential and/or increased histone retention sites in sperm	Increased differential noncoding RNA levels			3T3-L1 Mouse preadipocytes	Mouse, Rat	Increased Adipogenesis	(23–27)
*DDT*	Promotes DNA Methylation Regions		Promotes differential and/or increased histone retention sites in sperm	Pi-RNA and T-RNA fragments altered				Mouse	Increased Adipogenesis	(12, 24, 27, 28)
*DEHP*	Promotes DNA Methylation Regions							Rats, Drosophilia	Increased body weight	(9, 12)
*Bifenthrin*	Methylation at Promoter Region							Menidia beryllina	Enrichment of growth and development pathways	(10)
*Levonorgestrel*	Methylation at Promoter Region							Menidia beryllina	Enrichment of growth and development pathways	(10)
*Trenbolone*	Methylation at Promoter Region							Menidia beryllina	Enrichment of growth and development pathways	(10)
*BPA*	Methylation in promoter region	Transgenerational desilencing of H3K9/27^me3^ in germline						Mouse, C. elegans	Increased adipogenesis	(29, 30)
*TBT*	Biased DNA Methylation	Demethylates H3K27^me3^			Alteration in chromatin organization gene; alteration in higher order chromatin structure in MSCs, liver, and WAT; altered chromatin accessibility in adipogenesis associated genes		Mouse MSCs	Mouse, zebrafish	Predisposed to obesity	(31–33).
*Atrazine*	Promotes differentially methylated regions						Rat adipocytes			(34)

## DNA Methylation

DNA methylation most commonly occurs *via* transfer of a methyl group to the fifth carbon position of certain cytosine residues in the DNA sequence (35). DNA methylation is the most studied mechanism proposed to underlie the inherited effects of obesogen exposure. DNA methylation is a key epigenetic marker linked to regulation of gene expression, genomic imprinting, retrotransposon repression, and the inactivation of mammalian X-chromosomes (36, 37). DNA methylation typically influences gene expression by altering interactions between DNA, chromatin proteins, and specific transcription factors (38). DNA methylation may serve as a link between environmental exposures and heritable phenotypes. It has been suggested that epigenetic mutations (epi-mutations) such as DNA methylation can be inherited from one generation to another (5). For example, exposure of pregnant F0 rats to vinclozolin promoted the accumulation of differentially methylated regions (DMRs), in which the genomic DNA is methylated different cytosine residues in treated animals and their descendants compared with controls (39).

For inheritance of an environmental exposure to occur, the germline must be involved. It should be noted that due to the difficulties of isolating sufficient numbers of oocytes from female animal models, much of the molecular mechanisms involving epigenetics has been done on the male germline (39). Previous studies (23) found that exposure of pregnant F0 rat dams to vinclozolin led to DMRs in the F3 generation sperm and a different germ cell epigenome during different phases of F3 embryonic development between days 13 and 16. Analysis of the top 100 statistically significant differences in DNA methylation of the F3 generation after exposure to vinclozolin showed altered DNA methylation regions in the primordial germ cell (PGCs). These results could indicate that multiple DNA methylation alterations in PGCs could be a mechanism underlying transgenerational inheritance. In a separate study with vinclozolin, F1 and F2 generation rats showed minimal disease phenotypes while the F3 generation developed kidney disease as well as prostate and testes abnormalities, each with unique disease related DMRs (40). This same study reported sex-dependent transgenerational effects in F3 female mice for obesity.

Glyphosate is a controversial herbicide and well-known EDC. It was used in an *in vivo* study using rats to explore possible epigenetic changes that cause a variety of environmentally induced diseases. Glyphosate exposure to F0 females was found to affect unexposed F3 males with a variety of disease including kidney disease, prostate disease, and obesity, and females with various reproductive diseases and abnormalities (41). Analysis of DMRs and differential histone retention sites (DHRs) of F3 males revealed that certain DMRs and DHRs remained consistent across all diseases, while other DMRs and DHRs showed patterns specific to each unique pathology (42). That unique patterns of DMRs and DHRs corresponding to specific disease may exist indicates that epigenetic disease biomarkers could exist within sperm chromatin.

Epigenetic effects were seen in the adipocytes of F3 generation rats that were ancestrally exposed to DDT, atrazine, or vehicle control. DMRs were analyzed using MeDIP sequencing to identify overlapping and unique DNA methylation patters between each group. It was concluded that both DDT and atrazine promoted unique DNA methylation changes in male and female adipocytes of DDT exposed animals, suggesting that these were sex-dependent changes. In addition, exposure to both toxicants resulted in DMRs that differed from vehicle controls in both male and female adipocytes. The authors further reported that changes in DNA methylation were observed in approximately the same regions between lean and obese phenotypes that were associated with obesity, type 2 diabetes, and other metabolic syndromes, although the resolution of the method was insufficient to localize these changes to specific nucleotides or to distinguish between regions that were more methylated vs. less methylated (34). Rats exposed to DDT (11), jet fuel (JP-8) (43), BPA, DEHP, or dibutyl phthalate (DBP) (12), exhibited an increased incidence of adult-onset obesity, together with numerous other adverse effects on various tissues. In the 2013 study by Manikkam et al., in which mice were given a higher or lower dose of a plastic mixture of DHEP, BPA, and DBP, the F3 generation was most affected with reproductive abnormalities and obesity. Interestingly, F3 females experienced a very significant increase in ovarian disease over the F1 generation, indicating more robust effects in a later generation. It was suggested that these phenotypes were related to DMRs that may be associated with the epigenomic reprogramming of the male germline (12, 43). Gestating F0 rat dams were exposed to DDT or DMSO vehicle controls and epigenetic effects were tracked into the F3 generation. Comparison of DMRs between generations showed that most of the sperm DMRs were unique for each generation, that is the changes observed in one generation were not the same in other generations. It was proposed that the unique DMRs in each generation corresponded with the varying repertoires of disorders observed in different generations including obesity, ovarian, testes, kidney, and prostate disease (24). The minimal overlaps between generations shows that more studies must be done to strengthen this argument.

There is a genome-wide erasure of DNA methylation in mammals twice each generation, first during early zygotic development and again during the development of germ cells [reviewed in (44)]. Together with numerous observations (above) that specific DNA methylation patterns are not conserved within tissues or across generations it is difficult to conceptualize how DNA methylation can mediate transgenerational inheritance by itself. Some have expressed skepticism about the existence or prevalence of epigenetic transgenerational inheritance in mammals (45). However, independent evidence supporting mammalian transgenerational phenotypes has been accumulating for multiple stressors and model systems, establishing a solid foundation for this field of research [reviewed in (13)]. DMRs are observed in unexposed generations irrespective of how they occurred. Others showed that changes in DNA methylation observed in murine fetal germ cells after exposure to EDCs disappeared in the next generation as a likely consequence of global erasure of the epigenetic marks in primordial germ cells and zygotes (46, 47). However, erasure of DNA methylation by the processes of epigenetic reprogramming is not complete, and methylation of several types of genomic elements such as repetitive sequences escape erasure (48). Genomic imprinting is a special mode of gene expression where the parent-of-origin specific DMRs and other epigenetic marks dictate monoallelic expression of the homologous genes (49). Many imprinted genes escape epigenetic erasure in zygotes, although most of them are reprogrammed during germline cell development (49, 50). Thus, it remains possible that DNA methylation has a role to play in transgenerational inheritance. Such confirmation would require additional studies employing whole genome bisulfite sequencing to identify consistent alterations in DNA methylation across generations to establish a causal link between DNA-methylation and transgenerational inheritance.

## Histone Methylation

Post-translational modifications can occur on histone proteins in the nucleosome, which help maintain the tightly packed chromatin structure in DNA (37). Modifications at histone sites help control gene expression have been an area of interest in relation to stem cell lineage fate and adipogenesis (51, 52). Epigenetic changes in histone methylation, in which methyl groups are added to histone proteins to regulate transcription, have been found to play important roles in MSC and preadipocyte differentiation (52). Histones are commonly methylated on lysine, arginine, serine, and threonine residues (37).

Disruption of histone methylation in developing sperm impaired the health, development and survivability of offspring across multiple generations in mice (53). Kdm1a (lysine demethylase 1a) is a mouse histone lysine demethylase that controls gene expression and development by altering histone methylation (54). When a *kdm1a* transgene was over-expressed in mice, development of offspring was impaired, resulting in skin and bone abnormalities as well as stunted growth (53). This impaired development persisted for up to two generations after initial exposure to the transgene (53). These lingering effects of the transgene support the possibility that histone methylation is a possible molecular mechanism underlying paternal epigenetic inheritance (53).

Out of the four major types of nucleosomal histones (H2A, H2B, H3, and H4), histone H3 (H3), in particular, is a primary site of modification and has the most substantial amount of methylation sites when compared to the remaining histones (52, 55). The effects of histone methylation on gene expression depends largely on the location of the residue on the histone tail and degree of methylation or other modification. For example, while trimethylation of histone H3 on lysine 4 (H3K4me3) is typically associated with active transcription, trimethylation on lysine 27 (H3K27me3) is associated with transcriptional suppression (56). Repression of genes important for differentiated phenotypes is prominent in stem cells because this maintains the stemness of the cells and prevents them from differentiating into specific cell fates in the absence of stimulation. For MSCs to commit to the osteoblast lineage, adipogenic genes must be repressed by transcription factors that prepare the chromatin landscape for osteogenic stimulation and inhibition of adipogenesis (57). In contrast, in order for TBT to induce adipogenesis in MSCs, it must first de-repress genes involved in adipogenic commitment (31). Enhancer of zeste homolog 2 (Ezh2), is an H3K27 methyl transferase subunit that deposits trimethyl marks on H3K27. Downregulation of Ezh2 expression positively regulated adipogenesis and inhibited osteogenesis *via* H3K27me3 regulation at WNT gene promotors (58–61). Notably, TBT exposure in MSCs decreased the expression of EZH2, resulting in decreased deposition of repressive H3K27me3 marks near genes important for adipogenic commitment, which led to increased expression of mRNAs encoded by these genes (31). TBT also up-regulated expression of mRNAs associated with extracellular matrix proteins and lipid metabolism genes that regulated adipogenesis and adipocyte function in mouse MSCs, producing what were termed, unhealthy adipocytes (62). A similar effect of TBT was also shown using the 3T3-L1 preadipocyte model (25).

Another methyltransferase, histone H3 lysine 4 methyltransferase (H3K4MT) also impacted adipogenesis, *in vivo*. Mice deficient in H3K4MT were resistant to high fat diet (HFD) induced accumulation of WAT and hepatic lipid accumulation (fatty liver) (63). These mice also showed an increased insulin sensitivity and energy expenditure, demonstrating the importance of histone methylation on key metabolic endpoints (63). In more recent studies, demethylases such as JMJD2B were found to promote adipogenesis *via* stimulation of PPARγ and CEBPα binding proteins in HFD-fed mice (64).

Diet induced alterations in male mice who were given a folate rich or a folate deprived diet, were consistent with an underlying epigenetic transmission of methylation in sperm H3 methylation (65). Mice descended from males fed a low-folate diet exhibited aberrant expression of genes required for normal embryonic development, resulting in increased embryo loss and birth defects. Affected genes observed in sperm included *lhx6* and *ers1*, *tcf21*, and *prox1*, which correspond to central nervous system, kidney, and muscle development, respectively. Folate deficient males produced sperm that contained lowered levels of H3K4 and H3K9 methylation compared to folate sufficient males, suggesting that methylation was in some way dependent on diet. Because no significant DNA breaks were detected in sperm or their precursor cells, the developmental anomalies caused by paternal low folate diet were attributed to alterations in the sperm epigenome rather than to DNA damage (65). This supports a model in which transgenerational epigenetic transmission of the developmental abnormalities was caused by folate deficiency (65). Mice descended from males fed with a low-protein diet also showed impacts on metabolic outcomes such as upregulated lipid biosynthesis pathways. The sperm of mice siring these offspring exhibited a decrease in H3K27me3 at the monoamine oxidase A (*Maoa*) promoter (66). Female offspring of male mice fed with a HFD showed considerable changes in the transcriptomes of retroperitoneal WAT, involving about 5000 differentially expressed genes (67). Male *Drosophila melanogaster* fed a high sugar diet exhibited transcriptional depression in sperm that were dependent on the silencing of H3k9me3 and H3k27me3 (68). Taken together, these studies support a model in which environmental and lifestyle factors affect the health and development of future offspring *via* histone methylation.

Transgenerational inheritance of histone methylation has been explored in experiments evaluating the effects of ancestral exposure to BPA in *Caenorhabditis elegans*. BPA exposure resulted in a transgenerational de-silencing of an epigenetically silenced H3K9/27me3 transgene in the germline that persisted through 5 generations without further exposure (29). De-silencing of the transgene was observable as a significant increase in germline apoptosis and higher level of embryonic lethality in the F3 generations. This BPA exposure eventually becomes embryonically lethal to *C. elegans*, and acts by the downregulation of genes required for oocyte maturation and viability (69).

## Histone Retention

Whereas the role of histone methylation in epigenetic inheritance has been explored to some degree, much less attention has been given to histone retention sites. During the meiotic phase of spermatogenesis, haploid spermatids undergo chromatin restructuring, in which a degree of histone retention occurs naturally. In the process of histone retention, nucleosomes fail to replace histones with transition proteins, and then ultimately, with protamines (70–72). Histone replacement is thought to be protective in nature, as the protamines create a more compact and tightly wound chromatin structure, decreasing susceptibility to genetic mutations and to DNA damage (58). However, when histones are not replaced with protamines, histone retention occurs and those sections of DNA are not as tightly wound increasing susceptibility to genetic and epigenetic changes.

The spermatid genome and the morphological process of chromatin remodeling during spermiogenesis have been reviewed extensively, however, the exact mechanism and function of histone retention remains largely unknown [reviewed in (72–74)]. Altered histone retention in sperm after EDC exposure has become a topic of interest, with special attention having been given to the transgenerational effects on the F3 generation. Earlier research from the Skinner lab suggested that DNA methylation and changes in non-coding RNA (ncRNA) were two major avenues that could underlie epigenetic transgenerational phenotypes (26, 75). These findings were extended to explore the effects of EDCs on histone retention and epigenetic transgenerational inheritance. An *in vivo* rodent study involving exposure to the commonly used fungicide vinclozolin, and the currently banned pesticide, DDT (dichlorodiphenyltrichloroethane), resulted in excess H3 retention in F3 male decedents of F0 females that were exposed during gestation to DDT or vinclozolin (76). The increase in H3 retention sites were detected in addition to core histone retention sites which remained constant across the control and experimental rats. When ChIP-seq was used to compare the amount of histone retention to histone H3K27me3 modification between control vs DDT lineage rats, H3 retention was found to be more prevalent than histone methylation. These results indicated that environmentally induced changes by DDT in sperm cells involved three avenues of change: the alteration of histone retention sites, changes in DNA methylation, and changes in ncRNA (discussed below) that could lead to sperm-mediated inheritance of disease. Previous studies on rodents with vinclozolin exposure have found transgenerational disease inheritance to include the dysfunction of male fertility, prostate and kidney disease, and cancer, while DDT exposure caused transgenerationally-inherited increased white adipose depot size (77).

In a separate study where F0 dams were given vinclozolin, differential histone retention sites were found to increase in number from the F1 to the F3 generation of male offspring compared to control lineage rats (27). The discovery that histone retention sites were affected by EDCs and that this effect increased through the generations has added another promising mechanism to the etiology of environmentally induced epigenetic transgenerational inheritance of disease (24, 27).

While it is known that EDCs such as those mentioned above can also act as obesogens, it is notable that a high fat diet alone, without the presence of obesity inducing chemicals, has also been found to alter histone retention. Mice treated with a high-fat diet (HFD) vs. a low-fat diet (LFD) showed differences in H3 retention sites in sperm. Chromatin immunoprecipitation (ChIP) assays revealed that a HFD was associated with higher H3 retention in promotor regions, whereas LFD mice showed lower H3 retention and at coding regions. HFD-fed mice exhibited increased overall enrichment of histone H3 at genes involved in embryonic development, and differential H3K4me1 enrichment at genes with the GO terms transcription regulatory activity, transcription factor activity, sequence-specific DNA-binding, DNA binding, neuron differentiation and transcription (70). Expression of mRNAs encoding seven such genes (of 20 evaluated) was significantly altered in livers of HFD-fed male offspring compared with controls. ChIP-seq analysis demonstrated altered occupancy of H3 at genes known to be involved in embryonic development. Analysis of sperm histones revealed that H3K4me1 was enriched near transcriptional regulatory genes in males fed a HFD compared with controls, suggesting that retention of this histone mark was different in sperm of HFD-fed males and from the overall enrichment pattern of H3. The genes whose promoters was enriched in sperm from HFD-fed males included the transcriptional regulator *Lfd5*, the chromatin remodeling factor, *Lsh*, the homeobox-containing genes *Hoxd11* and *Hoxd13*, the forkhead-containing transcription factors *Foxp2* and *Foxa2* as well as the angiogenesis inhibitor *Bai3*. Thus, it is possible that differential occupancy of H3 as well as location of H3K4me1 retention sites play important roles in transmitting the effects of paternal HFD to offspring *via* the sperm epigenome leading to differential expression of a set of genes required for important processes in development (70).

Since the mechanism by which histone retention occurs remains unknown, recent research is further exploring different avenues through which transcription factors may play a role in histone retention and epigenetics (72). Other avenues that may lead to histone retention include chromatin remodeling, which will be further discussed, as well as the competitive binding of nucleosomes at CgP islands (72). CTCF and cohesin, two major proteins that help to modulate the 3D structure of chromatin, have been found to affect the binding of specific transcription factors and to be affected by transcription factor binding. It has been proposed that transcription factor binding can affect histone modification and may subsequently be important for epigenetic inheritance [(78, 79); reviewed in (80)]. While histone retention appears to be involved in transgenerational inheritance, there seems to be a driving force behind it that allows retention to occur.

## Small RNAs and RNA Fragments in the Male Germ Line

Another epigenetic mechanism possibly underlying transgenerational inheritance involve RNA molecules that do not translate into proteins, called noncoding RNAs (ncRNAs) in the mammalian male germline. Noncoding RNAs, including short RNAs, which include microRNA (miRNA) and piwi-interacting RNA (piRNA) and longer RNAs such as long non-coding RNA (lncRNA), have all been found to play a role in changing gene expression (81). Adipose tissue is known to be a hotspot for miRNA, which degrades mRNA molecules and prevents certain genes from being transcribed, linking it to metabolic disfunction and obesity in animals and humans (81).

ncRNA in germ cells are thought to play a role in epigenetic changes that are linked to obesity. When sperm exit the testis and move from the proximal to distal epididymis, their major cytosolic RNA species shifts from piRNAs to tRNA fragments. These tRNA fragments may be partly delivered by epididymosomes, small vesicles secreted by the epididymal epithelium (82). Conine et al., generated mouse zygotes using intracytoplasmic injection of sperm collected from the proximal part of the epididymis and observed that these zygotes were not competent to implant due to the overexpression of multiple epigenetic regulators such as RNA binding proteins and chromatin-associated factors (83). However, injecting small RNAs specific to sperm in the distal epididymis into the faulty zygotes rescued competence to implant (83). These data support a crucial role for tRNA fragments delivered through epididymosomes for post-testicular maturation of sperm. It also raises the possibility of soma to germ line transfer of ncRNAs. Whether this mechanism plays a significant role in epigenetic transgenerational inheritance needs to be elucidated in future studies.

Obesogen exposure can alter expression of noncoding RNAs in the offspring. Vinclozolin or DDT exposed rats had significantly higher expression of both lncRNAs and short ncRNAs (sncRNAs) in the ovarian granulosa cells of the F3 generation compared to controls (84). Furthermore, over 200 differentially expressed small ncRNAs were found in granulosa cells of both vinclozolin- and DDT-exposed rats and these noncoding RNAs were associated with previously observed obesogen induced disease phenotypes (26). MicroRNA makeup of paternal sperm have also been shown to affect the offspring phenotype. Injection of microRNAs gathered from the testes or sperm RNA into male mice that were fed a high fat diet led to metabolic disorders and altered expression of metabolic pathway genes in the offspring, whereas RNAs gathered from controls male mice did not (9, 85). Furthermore, consumption of high fat diet by pregnant F0 generation mouse dams led to defects in glucose and lipid metabolism in the F1, F2 and F3 generation (86). RNA-seq analysis of small RNAs of the F1 males showed large changes in the small RNA and tRNA-derived fragments, raising the possibility that noncoding RNAs could be a driving force of the non-genetic transgenerational inheritance. A similar study showed that high fat diet up or downregulated three sperm-borne microRNAs and passed the obesity phenotypes to the offspring. Although same phenotypes were also observed in the F2 as well, there was no evidence that same micro RNAs were dysregulated. This indicates that interaction with other mechanisms with microRNAs might act between F2 and F3 generation (87). Combined these data indicate that obesogens may change the makeup of the noncoding RNAs in the offspring and that such epigenetic changes might be associated with the non-genetic inheritance of disease traits. Noncoding RNAs have also been shown to serve as epigenetic regulators by interacting directly with DNA methylation, histone modification and chromatin remodeling (88). It has been suggested that such interactions may contribute to the non-genetic inheritance as well (89). At this point, the potential mechanisms underlying these phenomena remain under-studied.

Studying tissue or cell specific ncRNAs comes with challenges that make it difficult to determine the extent to which they contribute to inheritable epigenetic changes. Much about the mechanism of ncRNA action remains unknown and it is likely that other unknown functional elements are involved in ncRNA function. One major challenge in studying the heritability of ncRNA is that the sequences in these regions are not conserved, making it difficult to determine if there is a syntenic region within the organism (90).

## Chromatin Structure

The epigenetic mechanisms described above have gained experimental support for their involvement in transgenerational epigenetic inheritance, yet they each contain shortcomings. DNA methylation is erased genome-wide twice each generation so how can DMRs be observed in the F3 generation and beyond? The mechanisms through which histone methylation can be transmitted across generations or why certain histones are retained during spermatogenesis remain unknown. While there is evidence that ncRNAs can be found in sperm and seminal fluid to influence inheritance, it is unclear how their expression is transmitted to multiple future generations.

We recently presented a reproducible animal experimental system of transgenerational epigenetic inheritance in which exposure of pregnant F0 mouse dams to TBT throughout pregnancy and lactation led to a transgenerational “thrifty” phenotype. That is, F4 generation males (but not females) descended from exposed F0 dams gained considerably more WAT when dietary fat was increased and these F4 males resisted losing this fat after diet was returned to normal chow (21). The affected F4 males also resisted losing fat during conditions of fasting whereas F4 females or F4 male descendants of non-exposed control F4 dams did not (21). This is reminiscent of the situation in obese humans where fat is easy to gain and hard to lose.

Whereas numerous DMRs were identified in the F4 WAT, these DMRs showed no overlap with the promoters of differentially expressed genes (DEGs). Instead, DEGS in F4 male WAT were associated with large blocks of gDNA characterized by concurrent increases or decreases in DNA methylation. We denoted these gDNA segments iso-directional differentially methylated blocks (isoDMBs), which strongly linked DEGS with altered DNA methylation (21). Intriguingly hypomethylated isoDMBs were enriched in metabolic genes such as leptin, were located in chromosome regions relatively high in GC compared with AT and these regions are already known from other studies to correspond to topologically associated domains and loops (21). We noticed a strong correspondence between hypomethylated isoDMBs in F4 male WAT and inaccessibility of these regions in F3 and F4 sperm as measured by ATAC-seq in the ancestrally TBT-exposed group. In contrast, the hypermethylated isoDMBs in F4 male WAT were associated with a paucity of genes involved in metabolism, were GC-poor and were more accessible in F3 and F4 sperm of the TBT group. Based on these observations, we inferred that ancestral TBT exposure may have resulted in heritable changes in the higher order chromatin structure that led to increased expression of adipogenic and metabolic genes in WAT (21).

In a follow-up study using materials obtained from the same animals, analysis of DNA-methylation and transcriptome was studied in eight tissue samples in F3 and F4 mice. These eight samples included F3 and F4 male and female MSCs, F3 and F4 sperm, and F4 male liver and WAT. We observed that the ancestrally TBT-exposed group exhibited concerted alterations in the expression of genes related to chromatin organization and chromosome organization, suggesting that these changes were stable across mitosis and meiosis (32). Moreover these genes, together with those with the gene ontology term, metabolic processes were located in GC-rich isoDMBs in both the mouse and human genomes. The observation that chromatin and chromosome organization genes are located in GC-rich isoDMBs in multiple generations led us to hypothesis that chromatin structure was disrupted by ancestral TBT exposure and then either propagated to subsequent generations or that the disrupted structure was recreated every generation, much as normal structure is recreated in control animals (32). Based on these observations we proposed a new hypothesis that the non-genetic propagation of phenotypes across the generations relied on alterations in chromatin structure. Altered chromatin structure necessarily changes the accessibility of DNA and histones to modifying enzymes such as DNA and histone methyl transferases, the location and retention of histones and the expression of various genes, including those for non-coding RNAs. These mechanisms might interact and be preserved across generations and in various types of differentiated cells (32). In accordance with this hypothesis, exposure of zebrafish to low levels of TBT led to increased lipid accumulation and altered DNA accessibility at 349 chromatin regions at the H3K27me3 loci near genes involved in adipogenesis and metabolism (33). The epigenetic changes that took place in the chromatin of zebra fish larvae were proposed to prime the chromatin in early development, which led to changes gene expression and lipid accumulation later in life throughout development.

Exposure of pregnant F0 mouse dams to BPA has been shown to alter binding of the chromatin cohesion complex, CTCF, at several genomic sites. When pregnant F0 mouse dams were exposed to BPA, the obesity phenotype was inherited through the F6 generation offspring depending on the status of CTCF. The authors analyzed F1-F6 generation sperm and concluded that the development of obesity was dependent on the binding of CTCF at two enhancer regions of the *Fto* gene and that eliminating the CTCF binding site in this gene also eliminated the obese phenotype despite BPA exposure (91). Taken together, these results indicate that the chromatin structure model of transgenerational inheritance is plausible and requires further testing in mammalian models. Such studies are underway in our laboratories and elsewhere.

## Future Directions

There is still much to be learned about how the effects of obesogen exposure can be transmitted across generations. Although evidence for transgenerational inheritance of obesity after obesogen exposures is strong, only a definitive understanding of the underlying mechanisms will be critical for acceptance of the idea that the effects of environmental exposures can be inherited. Whereas each of the possible mechanisms discussed above has some plausibility of contributing to transgenerational inheritance, the actual impact of each mechanism and the extent to which it contributes to transgenerational inheritance remains unclear. The roles of transcription factors whose expression may be altered by environmental exposures in the transgenerational modulation of DNA methylation need further examination. A tantalizing but unproven possibility is that alterations in higher-order chromatin structure may underlie the epigenetic inheritance. In this model, changes in large-scale chromatin structures would allow or promote differential DNA methylation, histone methylation and histone retention, as well as expression and/or deposition of non-coding RNAs. Future studies in this field will be required to integrate preceding examples of epigenetic inheritance in mammals and proposed mechanisms into a comprehensive view so that the strength of contributions from each epigenetic mechanism to the non-genetic heritability of acquired traits can be objectively evaluated. This will allow us to distinguish between major driving forces in epigenetic transgenerational inheritance and fine-tuning modifiers.

## Author Contributions

EJ, NM, JS, BB, and TS wrote the paper. BB and NM edited and submitted the manuscript. All authors contributed to the article and approved the submitted version.

## Funding

Supported by grants from the National Institutes of Health, USA (R01ES023316 and ES031139) to BB and TS.

## Conflict of Interest

The authors declare that the research was conducted in the absence of any commercial or financial relationships that could be construed as a potential conflict of interest.

## Publisher’s Note

All claims expressed in this article are solely those of the authors and do not necessarily represent those of their affiliated organizations, or those of the publisher, the editors and the reviewers. Any product that may be evaluated in this article, or claim that may be made by its manufacturer, is not guaranteed or endorsed by the publisher.

## Abbreviations

ATAC-seq, assay for transposase accessible chromatin deep sequencing; BPA, bisphenol A; BPS, bisphenol S; ChIP-seq, chromatin immunoprecipitation deep sequencing; DBT, dibutyl phthalate; DDT, dichlorodiphenyltrichloroethane; DEG, differentially expressed gene; DEHP, diethylhexyl phthalate; DHR, differential histone retention site; DMR, differentially methylated region; EDC, endocrine disrupting chemical; gDNA, genomic DNA; GO, gene ontology; H3K4, histone H3, lysine 4; H3K27, histone H3, lysine 27; HFD, high fat diet; isoDMBs, isodirectional differentially methylated block; LFD, low fat diet; lncRNA, long non-coding RNA; miRNA, micro RNA; MSC, multipotent mesenchymal stromal stem cell, a.k.a. mesenchymal stem cell; ncRNA, non-coding RNA; piRNA, piwi-interacting RNA; PPARγ, peroxisome proliferator activated receptor gamma; RXR, retinoid X receptor, also known as 9-cis retinoic acid receptor; TBT, tributyltin; TPT, triphenyltin; VAT, visceral adipose tissue; WAT, white adipose tissue.
